# Distinct human skeletal muscle-derived CD90 progenitor subsets for myo-fibro-adipogenic disease modeling and treatment in multiplexed conditions

**DOI:** 10.3389/fcell.2023.1173794

**Published:** 2023-04-18

**Authors:** Angela Li, Madhavan Anbuchelvan, Amir Fathi, Maya Abu-Zahra, Denis Evseenko, Frank A. Petrigliano, Ayelet Dar

**Affiliations:** ^1^ Department of Orthopaedic Surgery, Keck School of Medicine, University of Southern California, Los Angeles, CA, United States; ^2^ Department of Stem Cell Research and Regenerative Medicine, University of Southern California, Los Angeles, CA, United States

**Keywords:** human muscle mesenchymal subsets, skeletal muscle differentiation, CD90, gp130 signaling, myo-fibro-adipogenesis, *in vitro* drug screening

## Abstract

Chronic muscle injuries, such as massive rotator cuff tears, are associated with progressive muscle wasting, fibrotic scarring, and intramuscular fat accumulation. While progenitor cell subsets are usually studied in culture conditions that drive either myogenic, fibrogenic, or adipogenic differentiation, it is still unknown how combined myo-fibro-adipogenic signals, which are expected to occur *in vivo*, modulate progenitor differentiation. We therefore evaluated the differentiation potential of retrospectively generated subsets of primary human muscle mesenchymal progenitors in multiplexed conditions in the presence or absence of 423F drug, a modulator of gp130 signaling. We identified a novel CD90^+^CD56^−^ non-adipogenic progenitor subset that maintained a lack of adipogenic potential in single and multiplexed myo-fibro-adipogenic culture conditions. CD90^−^CD56^−^ demarcated fibro-adipogenic progenitors (FAP) and CD56^+^CD90^+^ progenitors were typified as myogenic. These human muscle subsets exhibited varying degrees of intrinsically regulated differentiation in single and mixed induction cultures. Modulation of gp130 signaling via 423F drug mediated muscle progenitor differentiation in a dose-, induction-, and cell subset-dependent manner and markedly decreased fibro-adipogenesis of CD90^−^CD56^−^ FAP. Conversely, 423F promoted myogenesis of CD56^+^CD90^+^ myogenic subset, indicated by increased myotube diameter and number of nuclei per myotube. 423F treatment eliminated FAP-derived mature adipocytes from mixed adipocytes-FAP cultures but did not modify the growth of non-differentiated FAP in these cultures. Collectively, these data demonstrate that capability of myogenic, fibrogenic, or adipogenic differentiation is largely dependent on the intrinsic features of cultured subsets, and that the degree of lineage differentiation varies when signals are multiplexed. Moreover, our tests performed in primary human muscle cultures reveal and confirm the potential triple-therapeutic effects of 423F drug which simultaneously attenuates degenerative fibrosis, fat accumulation and promotes myo-regeneration.

## Introduction

The remarkable capacity of skeletal muscle to regenerate following injury can be severely impaired due to massive muscle wasting caused by repetitive trauma, tendon tears, and chronic injury (Testa et al., 2020; Subhash et al., 2022). Rotator cuff (RC) disorders are characterized by prolonged damage to shoulder tendons and muscles. The pathogenesis of injury-induced RC disease begins with a primary RC tendon tear, followed by a secondary progressive injury that develops in the shoulder muscles attached to the tendon as a consequence of musculotendinous retraction and subsequent forced uncoupling. Pathologic changes progressively develop in chronically damaged muscles and result in muscle scarring and wasting due to myofiber atrophy (Shah et al., 2017; Bogdanov et al., 2021; Subhash et al., 2022). Notably, one major factor that sets shoulder muscle tear injuries apart from other forms of skeletal muscle injuries is the massive accumulation of fat tissue in the injured RC (Davies et al., 2015). Cumulative studies provide evidence that mouse and human muscle-resident Gli1^+^ and PDGFRα^+^ interstitial cells are the origin of pathological fibrosis and adipose tissue (El Agha et al., 2017; Murray et al., 2017; Jensen et al., 2018; Theret et al., 2021) and are hence termed fibro-adipogenic progenitors (FAP). While injury activated FAP are responsible for muscle degeneration, myogenic progenitors robustly proliferate in response to myogenic cues in attempt to repair the damaged muscle (Theret et al., 2021; Loreti and Sacco, 2022). Recently, distinct non-myogenic PDGFRα^+^ subpopulations were identified in mouse muscle tissue by RNA-sequencing and were shown to respond differently to acute and chronic muscle injury (Leinroth et al., 2022). Still, human muscle progenitor studies are exceedingly limited in comparison to matched studies in mice due to a shortage in human muscle biopsies, *in vitro* modifications in marker expression and cell phenotype, as well as donor to donor variability. Therefore, human skeletal muscle has been modeled using primary immortalized myoblasts and mesodermal precursors or pluripotent cell-derived myogenic cells. However, primary cell lines acquire abnormalities in long term cultures and human pluripotent cell derivatives are developmentally immature. Thus, human muscle primary progenitors are still the best cell source for generation of cell-based reporter systems (Broer et al., 2020). Often, the expression of distinctive markers that typify distinguished tissue incorporated cell subsets rapidly changes in cultures post cell isolation. For example, the mesodermal precursors, perivascular adventitial cells, lose the expression of CD34 in cultures (Corselli et al., 2012) while CD56^+^ myogenic progenitors acquire high expression of CD90 at passage 0 (Testa et al., 2020). We therefore tested an alternative strategy for identifying different cell populations that can be utilized as platforms for drug testing in cultures to complement pre-clinical animal studies. For that purpose, human muscle-derived primary cell subsets were retrospectively defined and characterized based on marker combinations that were acquired and stably maintained in highly defined culture conditions.

Multiple factors have been implicated as regulators of regenerative and degenerative muscle remodeling, including fibrogenic factors such as TGFβ-1, IL-4, and IL-15 (Henderson et al., 2020; Theret et al., 2021) and adipogenic inducers such as UCP1, FGF-2, and Annexin A2 (Wang et al., 2018a; Wang et al., 2018b; Hogarth et al., 2019; Camps et al., 2020; Bogdanov et al., 2021; Mathes et al., 2021). Myogenic regulators are also involved, consisting of inflammatory factors IL-6, IL-4, IL-10, TNFα, and IL-1β, as well as factors secreted from muscle-residing cells such as WISP-1 (Lukjanenko et al., 2019; Loreti and Sacco, 2022). Of particular interest, the transcription factor STAT3 regulates multiple physiological changes in muscle and its effects are dependent on cell type, kinetics and duration of its activation, specific stage of muscle remodeling, injury settings, age, and genetic background (Wang et al., 2008; Mashili et al., 2013; Price et al., 2014; Tierney et al., 2014; Gopinath, 2017; Madaro et al., 2018; Sala et al., 2019). In accordance, several reports demonstrate that drug-induced modulation of STAT3 activation and its target genes prevent muscle wasting mainly in models of cancer cachexia (Sala et al., 2019) and attenuate denervation induced muscle atrophy (Madaro et al., 2018; Huang et al., 2020) and fibrosis (Madaro et al., 2018). Administration of antibodies against IL-6 receptors reduced STAT3 activation in mouse models of severe dystrophy and promoted improved muscle regeneration and inhibition of fibrosis (Wada et al., 2017). Nevertheless, much less is known about the involvement of STAT3 in the development and maintenance of abnormal muscle adipose tissue, and STAT3-dependent adipose wasting in a mouse model of cancer cachexia has only recently been described (Gandhi et al., 2022). Combined, these studies suggest that transient drug induced modulation of muscle precursor differentiation can potentially modify the progression of chronic muscle disease, affecting the simultaneously occurring processes of muscle fibro-adipogenic degeneration and regenerative myogenesis. We therefore studied the potential pleiotropic effects of gp130 signaling on myo-fibro-adipogenic differentiation of human muscle progenitor cells using 423F, an analog of a regulator of cartilage growth and differentiation (RCGD423) which was previously shown to mediate cartilage repair via modulation of gp130 signaling (Shkhyan et al., 2018).

As an innovative approach to study drug-induced modulation of muscle progenitor therapy, we first developed a reproducible protocol for prospective selection of discrete subsets of human muscle cells and identified a novel CD90^+^CD56^−^ non-adipogenic/markedly less fibrogenic cell subset. Secondly, we characterized how 423F-induced modulation of gp130 signaling, which was previously characterized in a mouse model for cartilage repair (Shkhyan et al., 2018), modifies human muscle progenitor lineage decisions in classic single and multiplexed myo-fibro-adipogenic induction cultures. Our studies combine a human muscle-derived myo-fibro-adipogenic multiplexed reporter system with matched drug screening in mixed conditions in order to better mimic *in vivo* conditions and predict pre-clinical responses *in vivo*. We found that the various cell subsets maintained a stable characteristic differentiation potential of either myogenic, fibro-adipogenic, non-adipogenic, or none at single and various combinations of myo-fibro-adipogenic inductions that only changed the degree of subset differentiation. Treatment with 423F diversly affected progenitor differentiation, markedly inhibiting FAP fibro-adipogenesis and significantly increasing myogenesis of the CD56 myogenic subset only. Moreover, our tests performed in primary human muscle cultures indicate that 423F drug could be potentially more effective for treatment of chronic muscle diseases that are associated with both fibrosis and fat accumulation without worsening muscle wasting or disrupting myo-regeneration.

## Materials and methods

### Human muscle biopsies and muscle cell cultures

Biopsies of healthy human muscle (n = 12, Supplementary Table S1) were obtained with informed consent and approved by the University of Southern California IRB. Healthy and diseased human muscle biopsies (Supplementary Table S1) were fixed in 4% formalin (Thermo Fisher Scientific) for histological and immunohistochemical analyses. Muscles were dissected into 1–2 mm diameter pieces and fragments were digested with 0.5 mg/ml Collagenase IV (Millipore Sigma) at 37°C for 40 min on a microplate shaker (Fisherbrand). Cell-containing supernatant was filtered through a 70 μm cell strainer (Fisherbrand). After centrifugation (Allegra X-14R, Beckman Coulter), cell pellet was resuspended in Endothelial Cell Growth Medium-2 (EGM-2, Lonza) and seeded into uncoated culture dishes (Corning) or Matrigel Basement Membrane Matrix-coated culture dishes (0.09 mg/ml, Corning). Pre-plating cultures were prepared by seeding human muscle fragments into uncoated culture dishes, and outgrown cell clusters were cultured in EGM-2. Where indicated, confluent adherent cells were then removed (Trypsin-EDTA 0.25%, Corning) for sorting of either CD56^+^CD90^+^ or CD90^+^CD56^−^ cell subsets. For all experiments, primary muscle cells were used between passages 2-6.

### Histology and immunohistochemistry

Human muscle sections were stained with H&E for general tissue structure analysis or picrosirius RED for collagen expression according to manufacturer instructions (Abcam, Cambridge, UK). For fluorescence microscopy, stained muscle sections were labeled with primary antibodies overnight at 4°C and with secondary antibodies for 30 min at room temperature. The antibodies used for this study are listed in Supplementary Table S2. DAPI (Molecular Probes, 1:1000) was used for nuclei labeling. Images were acquired with a CKX53F3 Compact Cell Culture Microscope (Olympus, Oberkochen, Germany) and a Keyence BZ-X (Itasca, IL, United States).

### Flow cytometry and cell sorting

Human muscle cells from primary cultures (passages 0–6) were removed (Trypsin-EDTA 0.25%, Corning), washed in PBS (Corning), centrifuged (Allegra X-14R, Beckman Coulter), and labeled according to the manufacturer instructions. The antibodies used for this study are listed in Supplementary Table S2. Cell sorting was performed using FACS via an Aria III Flow Cytometer (BD Biosciences). Debris and dead cells were excluded according to forward and side scatter data. Analyses were carried out using an Attune flow cytometer (Thermo Fisher Scientific) and FlowJo v10.8.1.

### Differentiation and cytotoxicity assays

For adipogenic induction, cells were cultured in DMEM/10% FBS (Corning) supplemented with 1 μM dexamethasone, 1 μM insulin, 0.5 mM IBMX (all purchased from Millipore Sigma), and 1% pen/strep (Gibco). Adipogenic differentiation was assessed with Oil Red O staining of lipids according to manufacturer instructions (ScienCell Research Laboratories). For fibrogenic induction, cells were cultured in DMEM/10% FBS supplemented with 5 ng/ml human-TGFβ1 (R&D Systems). For myogenic induction, cells were cultured in DMEM supplemented with 20% Human Male AB Serum (Access Biologicals) and 1% pen/strep. For adipo-fibrogenic induction (AF), cells were cultured in DMEM/10% FBS supplemented with 5 ng/ml human-TGFβ1, μM dexamethasone, 1 μM insulin, 0.5 mM IBMX, and 1% pen/strep. For adipo-myogenic (AM) induction, cells were cultured in DMEM supplemented with 20% Human Male AB Serum, 1 μM dexamethasone, 1 μM insulin, 0.5 mM IBMX, and 1% pen/strep. For fibro-myogenic (FM) induction, cells were cultured in DMEM supplemented with 20% Human Male AB Serum and 5 ng/ml human-TGFβ1 and 1% pen. For adipo-fibro-myogenic (AFM) induction, cells were cultured in DMEM supplemented with 20% Human Male AB Serum, 1 μM dexamethasone, 1 μM insulin, 0.5 mM IBMX, 5 ng/ml human-TGFβ1, and 1% pen/strep. All differentiating cells were analyzed at 6 days post induction. Fibrogenic differentiation was quantified via picrosirius red staining of collagens I and III (Abcam). Collagen-bound picrosirius red dye was extracted by 0.1 M NaOH solution (Millipore Sigma), and collagen quantification was performed using spectrophotometry via a SpectraMax iD3 Microplate Reader (λ = 540 nm, Molecular Devices). Adipogenic differentiation was quantified by Oil Red O staining and red pixel intensity measurements by AdobePhotoshop version 24.4.2. For identification of myotubes, cultures were fixed with 4% PFA (Thermo Fisher Scientific) for 10 min at room temperature, washed in PBS, and labeled with nuclear DAPI and anti-human striated MyHC I. A MyHC I^+^ cell that contained >3 nuclei with typical morphology was considered a myotube. All differentiation conditions were also tested in the presence of 1 μM and 10 μM 423F. Cultures from Matrigel coated dishes were the source for CD56 subset for all differentiation experiments. Cultures from uncoated dishes (both cell suspension and pre-plating conditions) were the source for CD90^+^ and CD90^−^ subsets for all differentiation experiments. Cytotoxicity of 423F was measured by XTT assay according to manufacturer’s instructions (Biotium). Cells were cultured in the presence of 1, 10 and 30 μM 423F in DMEM/10% FBS and XTT mixture was added after 3 days in culture and incubated at 37°C for 2 h. All experiments were performed in triplicates.

### Western blot analysis

Cells were cultured in 6-well tissue culture plates (Corning) with EGM-2. Following cell expansion, cells were induced with the appropriate differentiation media for each condition and cultured for 24 h. After 24 h, the cultures were rinsed with 4°C PBS (Corning) and then put on ice. RIPA Lysis and Extraction Buffer (Pierce) and Protease and Phosphatase Inhibitor (Pierce) were added to the cultures and incubated on ice for an hour, and then scraped with a cell scraper (VWR International). Protein lysates were then collected into 1.5 ml microcentrifuge tubes (Bioland Scientific) and sonicated 3 times with 5-s pulses at a 50% power via a Fisherbrand™ Model 50 Sonic Dismembrator (Fisher Scientific). Protein concentrations were determined using the BCA Protein Assay (Pierce) according to manufacturer instructions and then mixed with 4x Laemmli Sample Buffer (Bio-Rad) and beta-mercaptoethanol (Millipore Sigma). Samples were boiled at 100°C for 5 min with a Digital Heating Drybath (Thermo Scientific) and then loaded into Mini-PROTEAN TGC Precast Gels (Bio-Rad) for SDS-PAGE that were ran at 130 V until completion using a Mini-PROTEAN Tetra Cell (Bio-Rad). Gels were then transferred onto a 0.2-μm nitrocellulose Trans-Blot Turbo Transfer Pack (Bio-Rad). The running buffer used for SDS-PAGE was 10x Tris/Glycine/SDS (Bio-Rad). Following the transfer process, the nitrocellulose membranes were blocked with EveryBlot Blocking Buffer (Bio-Rad) and incubated overnight with primary antibodies anti-STAT3, anti-pSTAT3, anti-beta-actin, and anti-Histone H3 (all diluted 1:1000 with blocking buffer and purchased from Cell Signaling Technologies). Following primary antibody incubation, membranes were rinsed with TBST (1L double distilled H_2_O, 29.25g NaCl, 20 ml 1 M Tris-HCl, 20 ml 10% Tween) and incubated with secondary antibody (31463 from Invitrogen) diluted 1:4000 with blocking buffer. Following secondary antibody incubation, membranes were washed with TBST and developed with Clarity Western ECL Max Blotting Substrate (Bio-Rad). All membranes were imaged using a ChemiDoc XRS+ (Bio-Rad) and quantified using Fiji ImageJ software.

### Statistics

Data are presented as mean ± SEM (unless otherwise indicated in figure legends). Statistical analysis was performed via GraphPad Prism software and Microsoft Excel using one-way ANOVA, Student’s *t* test, and Tukey’s *post hoc* multiple comparisons. *p* < 0.05 was considered statistically significant.

## Results

### Fibro-fatty degeneration of human skeletal muscle

**FIGURE 1:**
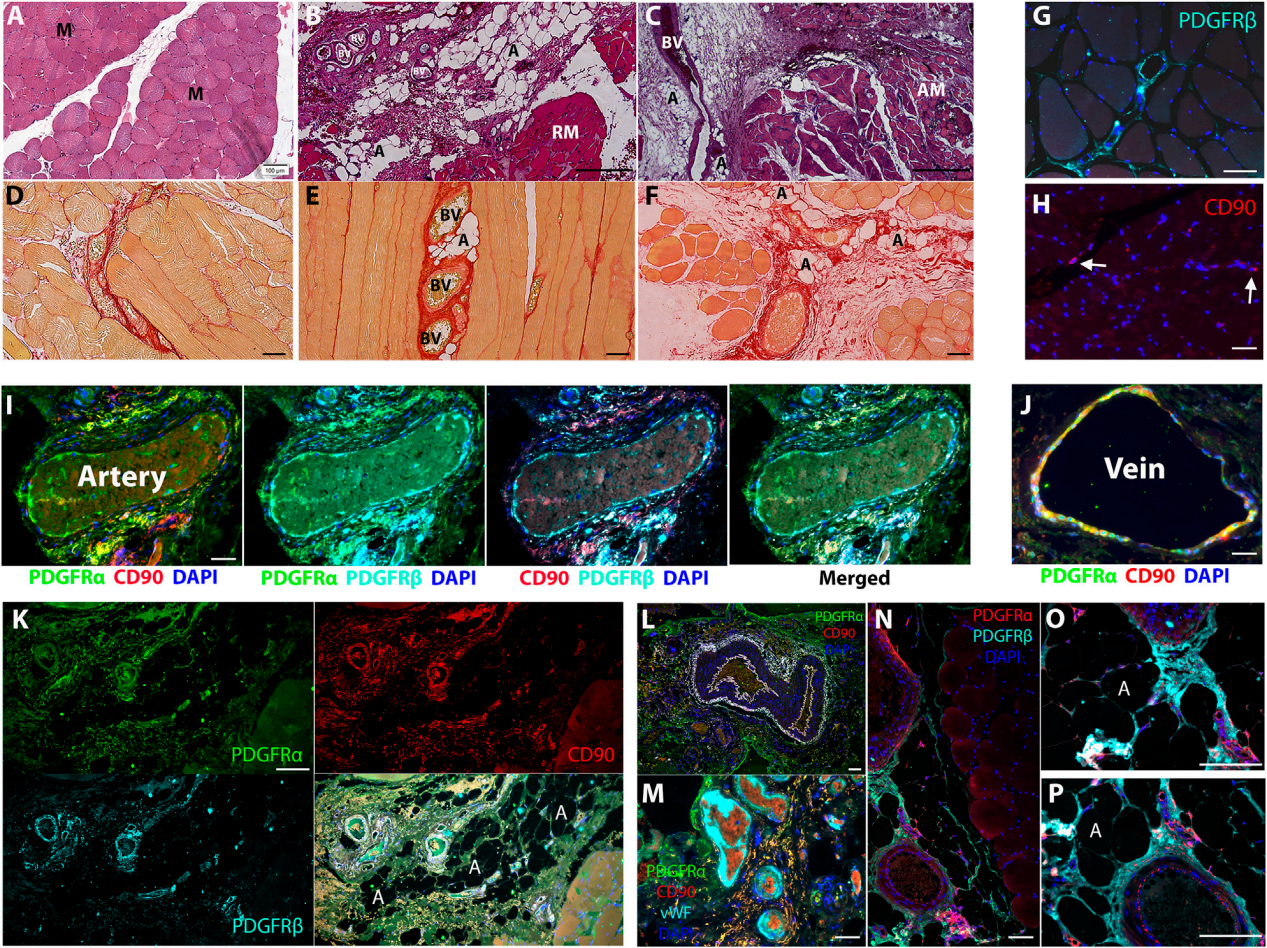
PDGFRβ^+^PDGFRα^+^CD90^+^ cells contribute to fibro-adipogenic lesion formation in diseased human muscle. **(A–C)** H&E staining of healthy **(A)** and diseased [**(B)**, moderate and **(C)**, severe] human muscle sections. Healthy myofibers (M) are replaced by adipose tissue (A) and fibrotic scars (F) that surround large blood vessels (BV) in muscle perimysium. Regenerating myofibers [**(B)**, RM] are substituted by smaller and amorphous atrophied myofibers [**(C)**, AM] with disease progression. **(D–F)** Picrosirius red staining for type I and type III collagen in healthy **(D)** early stage **(E)** and severe **(F)** disease stage. Small adipose tissue lesions are detected adjacent to collagenous blood vessels at early stage **(E)** and occupy larger areas close to fibrotic scars [**(F)**, red] at severe stage. **(G–P)** Representative images of healthy **(G–J)** and diseased **(K–P)** human muscle sections, immunolabeled for detection of PDGFRβ, PDGFRα and CD90 as indicated. Blue nuclear staining for DAPI. In healthy muscles the indicated markers are expressed by perivascular of arteries and veins **(G, I, J)** and interstitial cells [**(G, H)**, arrows]. PDGFRβ^+^PDGFRα^+^CD90^+^ cells occupy large fibrotic scars in interstitial spaces **(K)** and around large blood vessels **(I)** and form small lesions near arteries and veins in diseased muscles **(M–P)**. Blood vessel luminal endothelial cells were identified by the expression of von Willebrand factor and are seen surrounded by PDGFRα^+^CD90^+^ perivascular and scar residing cells **(M)**. Scale bars represent 100 μm.

In comparison to sections of healthy muscle biopsies (Figures 1A, D), diseased human RC muscles were characterized by loss of myofibers due to replacement with adipose tissue and fibrotic scars (Figures 1B–F). Small clusters of adipocytes were detected in proximity to large blood vessels (Figures 1B, E), and marked adipose tissue accumulation was observed next to massive vascular fibrosis (Figures 1C, F), implying perivascular cell origin of intramuscular fat and fibrosis. PDGFRα and CD90 were previously shown to demarcate FAP in diseased human muscle (Uezumi et al., 2014; Farup et al., 2021). In agreement, multiple other studies demonstrated that PDGFRβ and PDGFRα are strongly expressed by perivascular cells of various types of blood vessels, as well as interstitial cells in mouse and human muscles (Uezumi et al., 2014; Uezumi et al., 2016; Murray et al., 2017; Jensen et al., 2018). The combined expression of these markers in fibrotic muscle scars in early and late developmental stages is poorly defined, mainly due to the scarcity of biopsies from diseased human muscle. We therefore extended our analysis and mapped CD90, PDGFRβ, and PDGFRα co-expression in fibrotic lesions (Supplementary Table S1). As expected, PDGFRβ was highly expressed in healthy muscle by perivascular cells in all types of blood vessels as well as by perimysium and endomysium-residing interstitial cells (Figures 1G, I). In healthy muscle, CD90 expression was rarely detected in interstitial cells (Figure 1H) and was predominantly co-localized to PDGFRα^+^ perivascular cells of large veins (Figures 1I, J). All markers were dispersed between the three layers of the artery wall and were clearly co-expressed in the outermost layer, the tunica adventitia, as well as in the innermost layer, the tunica intima (Figure 1I). Next, we mapped the localization of PDGFRβ/PDGFRα/CD90 cells in fibro-adipogenic injured human RC muscles. The apparent development of massive fibrosis was accompanied by a robust increase in the number of PDGFRβ^+^PDGFRα^+^CD90^+^ cells, which populated the fibrotic scars (Figure 1K). Large fibrotic areas composed of PDGFRα^+^CD90^+^ cells were mostly located around large blood vessels that were identified by the presence of luminal red blood cells or von Willebrand factor^+^ endothelium (Figures 1L, M). Additionally, dense clusters of PDGFRβ^+^PDGFRα^+^ cells were seen emerging from the abluminal layer of perimysial blood vessels in areas of muscle undergoing fibro-adipogenic degeneration (Figures 1N–P). Taken together, these observations imply that PDGFRβ/PDGFRα/CD90 cell subsets are common drivers of fibro-adipogenic muscle disease with different etiologies.

### Generation of primary cell subsets from human muscle biopsies

**FIGURE 2:**
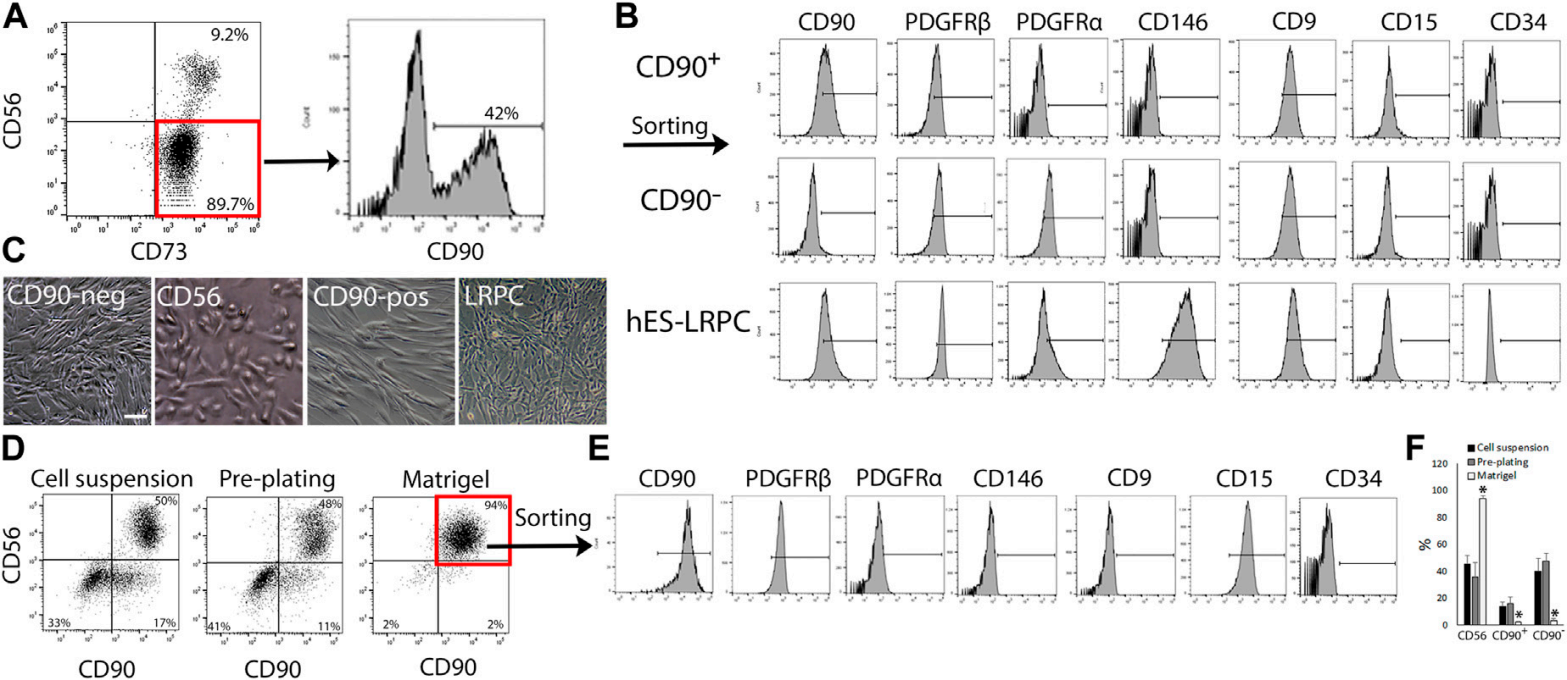
Identification and isolation of primary cell subsets from human muscles. **(A)** Representative flow cytometry analyses of CD73, CD56 and CD90 expression by primary human muscle cells cultured in EGM-2 medium at passage 0 (n = 8 donors). **(B)** Representative flow cytometry analysis of expression of characteristic cell surface markers: CD90, PDGFR-β, and CD146 (perivascular cells); CD9 (adipogenic progenitors), CD15 (myoblasts) and CD34 (endothelial, hematopoietic progenitors and adventitial cells) by either expanded human muscle cell sorted subsets (CD90^+^ and CD90^−^) or lineage restricted, perivascular-like cells (LRPC) that were derived from hES. Cells were analyzed between passages 1 and 5. **(C)** Representative images of cultured human muscle subsets and hES-LRPC. **(D)** Representative dot plots of tested conditions for human muscle myoblast enrichment and sorting strategy of myoblasts based on the expression of CD56 and CD90. Nearly all muscle cells were myoblasts when expanded in Matrigel coated dishes and EGM-2 medium at passage 0 (right panel). **(E)** Flow cytometry analysis of co-expression of perivascular markers, CD90, PDGFRβ and CD146 as well as the myoblast marker CD15 by sorted CD56^+^CD90^+^ human muscle-derived myoblasts between passages 1–3. **(F)** Frequency of CD56^+^, CD90^+^ and CD90^−^ subsets, harvested from uncoated culture dishes (cell suspension and pre-plating) or Matrigel coated dishes between passages 0 and 1. Data are mean ± SD, n = at least 3 donors per cell subset. Data analyzed via one-way ANOVA. *p < 0.001 between frequency of cell subset that was cultured on Matrigel, and the matched cell subset in cell suspension and pre-plating culture conditions. Scale bar represents 100 μm.

To gain better understanding on how human muscle cells respond to fibro-adipogenic-myogenic stimulus in injury settings, we sought to develop a stable primary cell subset platform that could maintain the phenotype of FAP and myo-progenitors. To confirm the reproducibility of the protocol for cell subset generation, biopsies were taken from a variety of healthy skeletal muscles, including subscapularis, deltoid, quadriceps, and pectoralis. A wide range of donor ages (between 20–81 years, n = 12) was tested as well (Supplementary Table S1). Cell composition was analyzed by flow cytometry when cultures reached 80%–90% confluency at passage 0. In agreement with previous studies (Uezumi et al., 2016; Goloviznina et al., 2020), all cell subsets gained and maintained high expression of CD73 in culture (Figure 2A). Three CD73^+^ cell subsets were consistently identified regardless of muscle type or donor age: 1. CD56^+^CD90^+^, 2. CD90^+^CD56^−^, and 3. CD90^−^CD56^−^ cells (Figures 2A–C; Supplementary Figure S1). Sorted CD56^−^ cell populations (Supplementary Figure S1) co-expressed PDGFRβ and CD9 and lacked the expression of CD146, CD15 and CD34. Only the sorted CD90^−^CD56^−^ subset (Supplementary Figure S1), but not the sorted CD90^+^CD56^−^ subset (Supplementary Figure S1), expressed the fibro-adipogenic marker PDGFRα (Figure 2B). We have previously published studies regarding lineage-restricted (non-myo-fibro-adipogenic) pericyte-like cells (LRPC) generated from human embryonic stem cells (Mosich et al., 2019) that were used as controls for our subset differentiation studies. Interestingly, LRPC exhibited a mixed-marker profile, expressing characteristic progenitor cell markers such as PDGFRβ and CD146 but lacking the expression of PDGFRα, a common FAP marker (Figure 2B). Tremendous enrichment in the frequency of cell populations that expressed the myogenic marker CD56 was achieved when culture dishes were coated with Matrigel in comparison to seeding of cell suspensions onto uncoated dishes or pre-plating of muscle fragments (Figures 2D, F). CD56^+^ subset dominated the culture at the end of P0 (Figure 2F) and flow cytometry analysis revealed that CD56^+^ myoblast-like cells acquired a marker expression profile, that is, more characteristic of perivascular/mesodermal cells but lack the expression of the fibro-adipogenic marker PDGFRα (Figure 2E).

### Mutable muscle subset fate under combined culture conditions characteristic of an injured muscle

**FIGURE 3:**
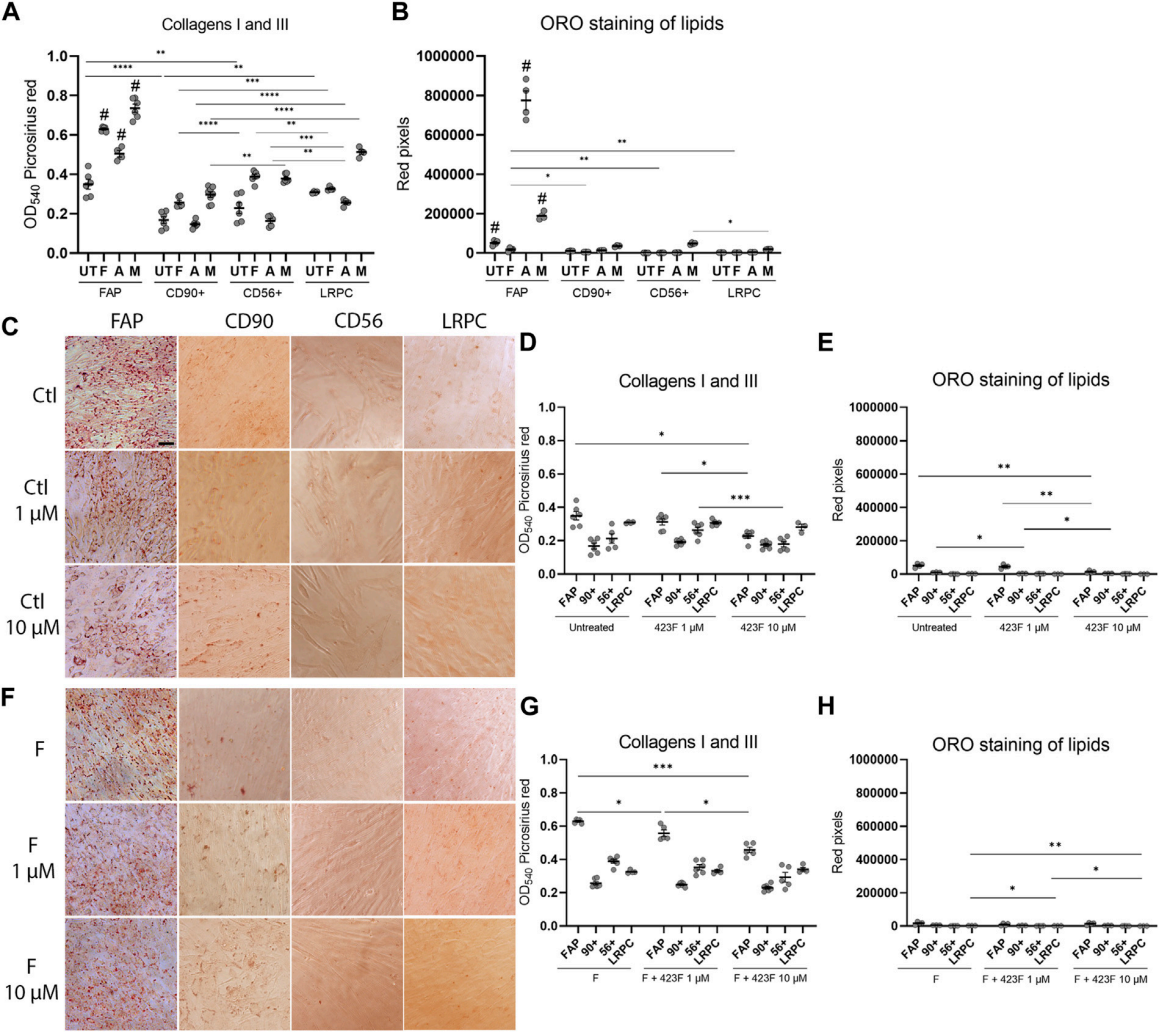
423F elicits greater inhibition of human muscle FAP fibro-adipogenic differentiation than that of CD56, CD90, and LRPC in control and fibrogenic cultures. Picrosirius red spectrophotometric quantification of collagen production **(A, D, G)** and red pixel quantification of Oil red O-stained adipocytes **(B, E, H)** at the indicated conditions (UT = untreated, F = fibrogenic, A = adipogenic, M = myogenic) in the presence or absence of 1 uM and 10 µM 423F. Data (mean ± SEM) was pooled from multiple experiments with triplicates for all cultures (n = at least 3 per cell subset). Representative images of Oil Red O-stained adipogenic cells in control **(C)** and fibrogenic cultures **(F)** for each cell subset. Data was analyzed via paired one-way ANOVA for comparisons between the same cell subset and unpaired one-way ANOVA for comparisons between different cell subsets, followed by Tukey’s *post hoc* multiple comparisons. # denotes significance between FAP and all other subsets in the matched culture conditions. #*p* < 0.0001, ****p < 0.0001, ***p < 0.001, **p < 0.01, *p < 0.05. Scale bar represents 100 μm.

**FIGURE 4:**
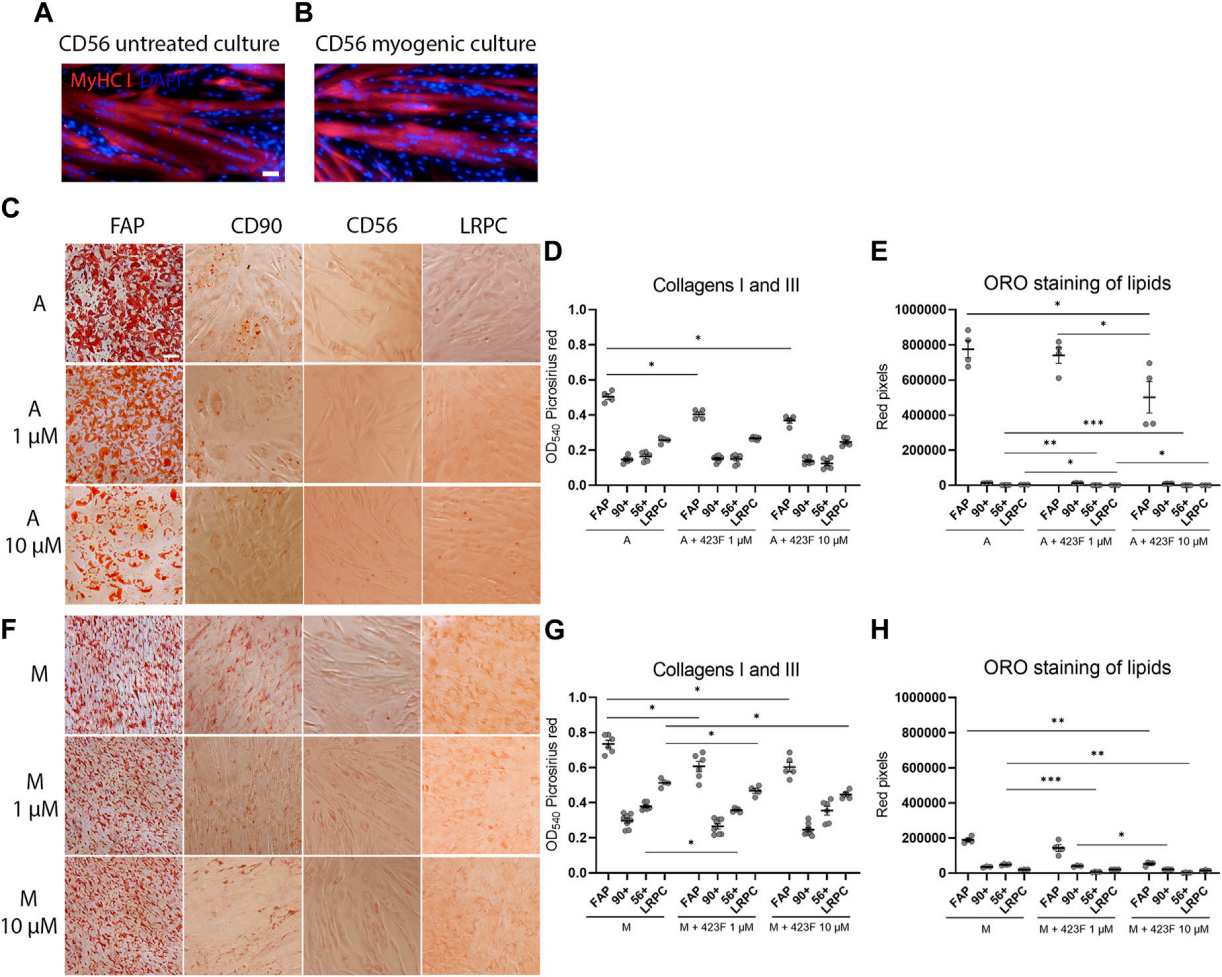
423F elicits greater inhibition of human muscle FAP fibro-adipogenic differentiation than that of CD56, CD90, and LRPC in adipogenic and myogenic cultures. **(A–B)** Myogenic differentiation is detected only in CD56 subset as seen in representative images of DAPI and Myosin heavy Chain I (MyHC I)-stained syncytial cells from CD56 in untreated culture **(A)** and myogenic serum-induced culture **(B)**. Representative images of Oil Red O-stained adipogenic cells in adipogenic **(C)** and myogenic cultures **(F)** for each cell subset. Picrosirius red spectrophotometric quantification of collagen production **(D, G)** and red pixel quantification of Oil red O-stained adipocytes **(E, H)** at the indicated conditions in the presence or absence of 1 uM and 10 µM 423F. Data (mean ± SEM) was pooled from multiple experiments with triplicates for all cultures (n = at least 3 per cell subset). Data was analyzed via paired one-way ANOVA and Tukey’s *post hoc* multiple comparisons. ***p < 0.001, **p < 0.01, *p < 0.05. Scale bar represents 100 μm.

**FIGURE 5:**
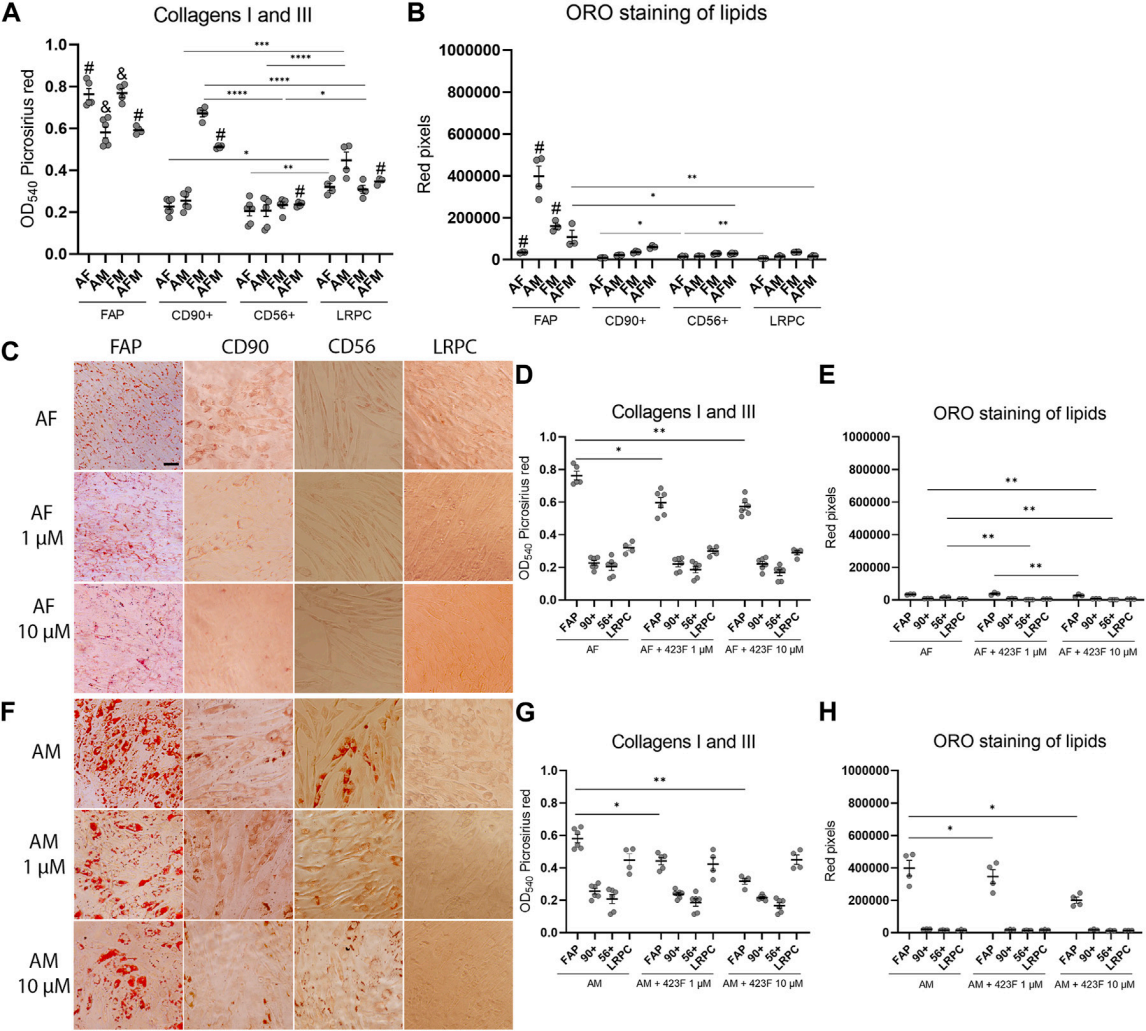
423F elicits greater inhibition of human muscle FAP fibro-adipogenic differentiation than that of CD56, CD90, and LRPC in adipo-fibrogenic and adipo-myogenic cultures. Picrosirius red spectrophotometric quantification of collagen production **(A, D, G)** and red pixel quantification of Oil red O-stained adipocytes **(B, E, H)** at the indicated conditions (AF = adipo-fibrogenic, AM = adipo-myogenic, FM = fibro-myogenic, AFM = adipo-fibro-myogenic) in the presence or absence of 1 and 10 µM 423F. Data (mean ± SEM) was pooled from multiple experiments with triplicates for all cultures (n = at least 3 per cell subset). Representative images of Oil Red O-stained adipogenic cells in adipo-fibrogenic **(C)** and adipo-myogenic **(F)** cultures for each cell subset. Data was analyzed via paired one-way ANOVA for comparisons between the same cell subset and unpaired one-way ANOVA for comparisons between different cell subsets, followed by Tukey’s *post hoc* multiple comparisons. # denotes significance between FAP and all other cell subsets in the AF condition, as well as significance between comparisons of all cell subsets in the AFM condition. & denotes significance between FAP and all other subsets in AM and FM conditions. #*p* < 0.0001, &*p* < 0.05, ****p < 0.0001, ***p < 0.001, **p < 0.01, *p < 0.05. Scale bar represents 100 μm.

**FIGURE 6:**
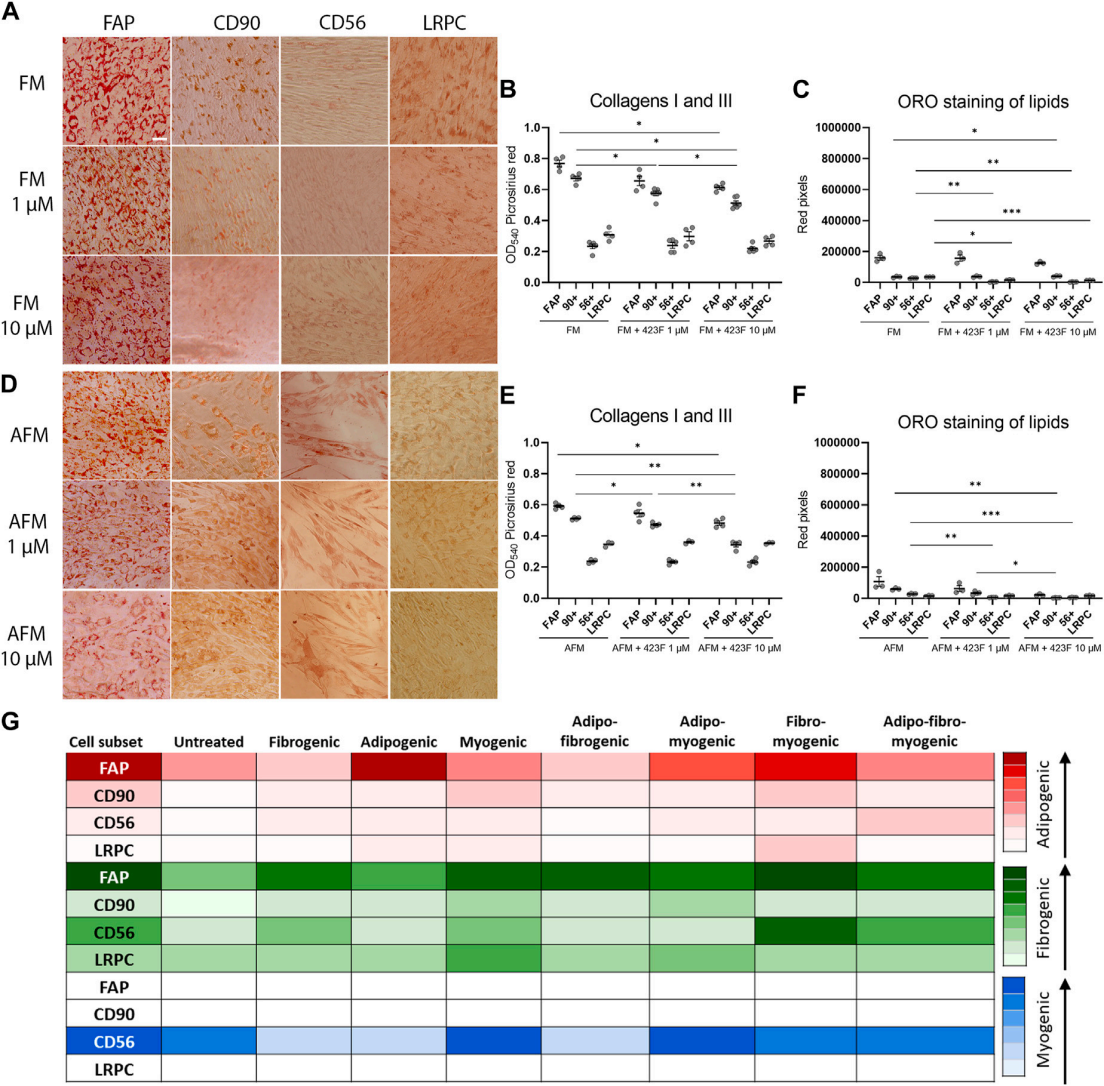
423F elicits greater inhibition of human muscle FAP fibro-adipogenic differentiation than that of CD56, CD90 and LRPC in fibro-myogenic and adipo-fibro-myogenic cultures. Representative images of Oil Red O-stained adipogenic cells in fibro-myogenic **(A)** and adipo-fibro-myogenic cultures **(B)** for each cell subset. Picrosirius red spectrophotometric quantification of collagen production **(B, E)** and red pixel quantification of Oil red O-stained adipocytes **(C, F)** at the indicated conditions in the presence or absence of 1 and 10 µM 423F. Data (mean ± SEM) was pooled from multiple experiments with triplicates for all cultures (n = at least 3 per cell subset). Data was analyzed via paired one-way ANOVA and Tukey’s *post hoc* multiple comparisons, ***p < 0.001, **p < 0.01, *p < 0.05. **(G)** Color scale denotes the degree of subset differentiation. Scale bar represents 100 μm.

We then further established the identity of each cell population by comprehensive evaluation of their differentiation potential using a broad differentiation culture system. To better mimic *in vivo* conditions, where cells simultaneously receive various signals with diverse regulatory effects, cell subsets were exposed to mixed culture induction conditions for characterization of cell fate decisions when interactions between various pathways take place. FAP (CD73^+^CD90^−^CD56^−^), CD90 (CD73^+^CD90^+^CD56^−^), and CD56 (CD73^+^CD56^+^CD90^+^) cell subsets, as well as LRPC (CD73^+^CD90^+^CD56^−^), were induced in either single fibrogenic (F), adipogenic (A), and myogenic (M) culture conditions, or mixed culture conditions at various combinations, including adipo-fibrogenic (AF), adipo-myogenic (AM), fibro-myogenic (FM) and adipo-fibro-myogenic (AFM) conditions. Following 6 days of culture, their fibrogenic and adipogenic responses were measured. In single-condition-induced cultures, FAP subset exhibited the highest fibrogenic collagen production in all tested conditions in comparison to CD56, CD90, and LRPC (Figures 3A, D, G, 4D, G). Exposure of prospectively isolated CD90 subset to adipogenic conditions defined it as a novel non-adipogenic subset with low fibrogenic potential and thus similar to LRPC (Figures 3B, 4C, E). Only FAP exhibited extensive adipogenic differentiation in adipogenic cultures (Figures 3B, 4C, E). FAP adipogenesis was mild in myogenic cultures and was abrogated in fibrogenic TGFβ-1-induced cultures (Figures 3B, F4C, E, F, H). FAP maintained high fibrogenic potential in all combined culture conditions that were slightly reduced in the presence of AM combinations, including AM and AFM (Figures 5A, D, G, 6B, E). AF (Figures 5B, C, E) and AFM (Figures 5B, 6D, F) inductions failed to rescue adipogenic differentiation of FAP, demonstrating a dual role of TGFβ-1 as a positive regulator of fibrosis and a negative regulator of adipogenesis.

As expected, CD56 (Uezumi et al., 2016; Goloviznina et al., 2020) and LRPC cells (Mosich et al., 2019) lacked adipogenic potential in all tested conditions (Figures 3B, C, E, F, H, 4C, E, 5B, C, E, F, H, 6A, C, D, F) except for a minimal adipogenic response observed for CD56 under AM and AFM conditions (Figures 5B, F, H, 6D, F). Unpredictably, in our system, CD90 subset did not demonstrate any magnitude of adipogenic potential either in single or mixed conditions (Figures 3B, C, E, F, H, 4C, E, 5B, C, E, F, H, 6A, C, D, F). Only CD56^+^ cells readily formed multinucleated, myosin heavy chain I (MyHC I) expressing myotubes in control and myogenic cultures (Figures 4A, B; Supplementary Figure S3). Increased number and size of MyHC I^+^ CD56-derived myotubes were measured in the presence of human-serum-enriched DMEM medium that was defined in accordance as myogenic medium (data not shown). Of notice, replacement of FBS with human serum evoked for the first time a functional response of LRPC, inducing an increase in collagen production (Figure 3A). This finding indicates that classical TGFβ-1 fibrogenic induction is sometimes not enough to reveal the fibrogenic features of certain cell populations. Overall, our differentiation studies reveal that cell fate is mainly governed by intrinsic features characteristic of each muscle cell subset with a mutable degree of differentiation provided by extrinsic signals (Figure 6G).

### Identification of 423F as an inhibitor of fibro-adipogenesis

Previous studies carried out in traditional single input differentiation cultures demonstrated that the gp130/STAT3 pathway plays a key role in the regulation of these differentiation pathways and is associated with intrinsic features of muscle cell populations (Wang et al., 2008; Price et al., 2014; Tierney et al., 2014; Madaro et al., 2018). We therefore elaborated upon this in our studies and evaluated the potential therapeutic effects induced by modulation of the gp130 pathway. For that purpose, we used the compound 423F, a structural analog of an experimental small-molecule drug (RCGD 423) that has been shown to target IL-6/STAT3 signaling and promote cartilage repair (Shkhyan et al., 2018). We first extended findings in human chondrocytes to human muscle cells and validated that 423F is neither cytotoxic nor an inducer of muscle cell differentiation (Figures 3C–E; Supplementary Figure S2). We found that 423F not only failed to induce cell differentiation, but it also inhibited minor FAP adipogenic differentiation and fibrotic collagen production in non-induced control cultures (Figures 3C–E). 423F decreased FAP adipogenesis in a dose-dependent manner in all single induction conditions that mediated FAP adipogenic differentiation, including untreated control and adipogenic cultures. Myogenic cultures (Figures 3C, E, 4C, E, F, H) induced further decrease in FAP adipogenesis in AM, FM, and AFM combined conditions (Figures 5H, 6C, F), and further attenuated the minimal adipogenic differentiation of FAP measured in AF cultures (Figure 5E). Spectrophotometric quantification of picrosirius red staining for collagens I and III revealed that 423F is also a potent inhibitor of fibrosis, as indicated by dose-dependent reductions in collagen production in the highly fibrogenic FAP subset, which was significant in most of the tested conditions (Figures 3D, G, 4D, G, 5D, G, 6D, G). Similarly, the presence of 423F in the much less fibrogenic subsets of CD90 and CD56 also reduced collagen production in fibrogenic cultures (Figure 3G). Predictably, the 423F-treated non-fibro-adipogenic LRPC, which served as non-differentiating control cells in this study, maintained basal levels of collagen production in comparison to untreated matched induced cultures at all tested combinations of inductions, except for increased collagen expression in the presence of human serum (myogenic cultures) (Figure 3A). Still, myogenic cultures did not induce myogenic differentiation of LRPC as indicated by the lack of multi-nucleated myotube formation (Figure 4F). Collectively, these data demonstrate that capability of myogenic, fibrogenic, or adipogenic differentiation is largely dependent on the intrinsic features of cultured subsets, and that the degree of lineage differentiation varies when signals are multiplexed (Figure 6G).

### 423F distinctively modulates STAT3 activation in differentiating cell subsets

**FIGURE 7:**
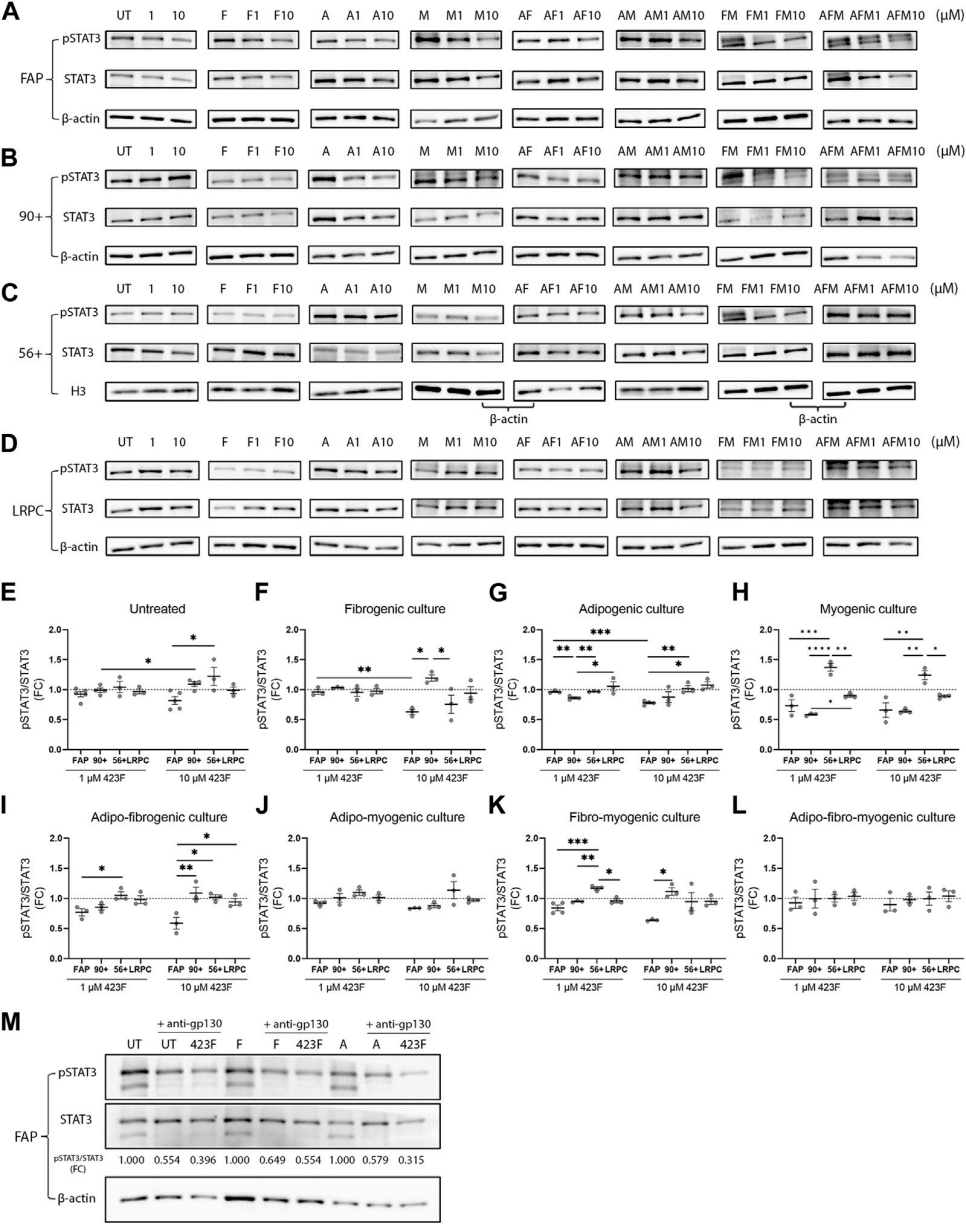
423F-mediated modulation of STAT3 expression and activity in cultures of human muscle cell subsets. **(A–D)** Representative images of western blot analyses of pSTAT3 and STAT3 from FAP **(A)**, CD90 **(B)**, CD56 **(C)**, and LRPC **(D)** whole-cell lysates harvested after 24-h in the indicated culture conditions. **(E–L)** Levels of pSTAT3 were related to total-STAT3 levels for each cell subset at the indicated conditions. Data (mean ± SEM) was pooled from multiple experiments and presented as fold change (FC) between indicated conditions not treated with 423F and indicated conditions treated with 1 μM and 10 µM 423F (n = at least 3 per cell subset, except for LRPC that were derived from a single cell source). Data was analyzed via paired one-way ANOVA for comparisons between the same cell subset and unpaired one-way ANOVA for comparisons between different cell subsets, followed by Tukey’s *post hoc* multiple comparisons. ***p < 0.001, **p < 0.01, *p < 0.05. **(M)** Representative images of western blots of pSTAT3 and STAT3 of control, 423F, and 423F and anti-gp130-treated FAP in untreated (UT), fibrogenic (F), and adipogenic (A) cultures.

The drug 423F is a gp130 modulator that acts via promoting gp130 homodimerization and preventing IL-6 and Oncostatin M-induced heterodimerization with IL-6R and OSMR respectively. In chondrocytes, 423F was shown to induce a marked increase in the levels of activated receptor-gp-130 (phosphorylated gp130) and in the levels of activated phosphorylated STAT3 (pSTAT3), the downstream effector. Given that STAT3 has been shown to regulate myogenic differentiation in a context-dependent manner (Wang et al., 2008; Price et al., 2014; Tierney et al., 2014) we further assessed its expression and activation in human muscle subsets in single and multiplexed myogenic, fibrogenic, and adipogenic culture conditions. The lineage-restricted hES-derived cell line was used to validate lack of activation of STAT3 in mesodermal cell counterparts. 423F mediated a slight dose-dependent decrease in STAT3 activation after 24 h in untreated cultures (Figures 7A, E). A stronger dose-dependent reduction in pSTAT3 was observed following induction of FAP differentiation in fibrogenic and adipogenic cultures (Figures 7A, F, G). This demonstrates that 423F/gp130 signaling antagonizes TGF-β1-mediated expression of collagen I and III, as well as insulin-induced adipogenesis, suggesting that 423F/gp130 signaling promotes downstream activation of SOCS3 that has been shown to negatively regulate gp130 (Carow and Rottenberg, 2014), TGF-β1 (Ogata et al., 2006), and insulin (Fan et al., 2014) signaling. Conversely, the myogenic CD56 subset exhibited higher levels of pSTAT3 in comparison to all other subsets in untreated culture (Figures 7C, E). There was a significantly greater dose-dependent increase in STAT3 activation in myogenic conditions (Figures 7C, H). In accordance, myogenesis took place only in untreated and myogenic cultures as indicated by detection of MyHC I^+^ multinucleated myotubes (Figures 4A, B). Like differentiating FAP, 423F inhibited STAT3 activation in differentiating CD56 in a dose-dependent manner (Figure 7H). Dose-dependent 423F/gp130-induced reduction in FAP-pSTAT3 could be still detected in AF and FM cultures (Figures 7A, I, K) but was abolished in AM and AFM cultures (Figures 7A, J, L). The non-adipogenic CD90 subset in turn exhibited opposite response to that of FAP and CD56, exhibiting a 423F/gp130-mediated dose-dependent increase in pSTAT3 in fibrogenic cultures (Figures 7B, F). The levels of pSTAT3 remained unchanged in adipogenic, myogenic, AM, and AFM conditions that did not elicit CD90 adipogenic differentiation nor significant changes in their 423F-promoted fibrogenic response (Figures 7B, G, H, J, L, 5G, H, 6E, F). FAP exhibited the highest sensitivity to 423F-mediated inhibition of STAT3 activation in comparison to CD56 and CD90 in matched culture conditions (Figure 7). As expected, the levels of pSTAT3 were not affected by 423F/gp130 signaling at all tested conditions and 423F concentrations (Figure 7D). To confirm that gp130 mediated 423F effects, FAP were co-treated with 423F and anti-gp130 in non-induced, fibrogenic, and adipogenic cultures. In all tested conditions, a greater reduction in pSTAT3 levels was detected in the presence of both 423F and anti-gp130 than that seen in the presence of 423F alone, validating previous data (Shkhyan et al., 2018) demonstrating that 423F acts through gp130.

### 423F/gp130 signaling elicits pleotropic effects on differentiated derivatives of muscle progenitors

**FIGURE 8:**
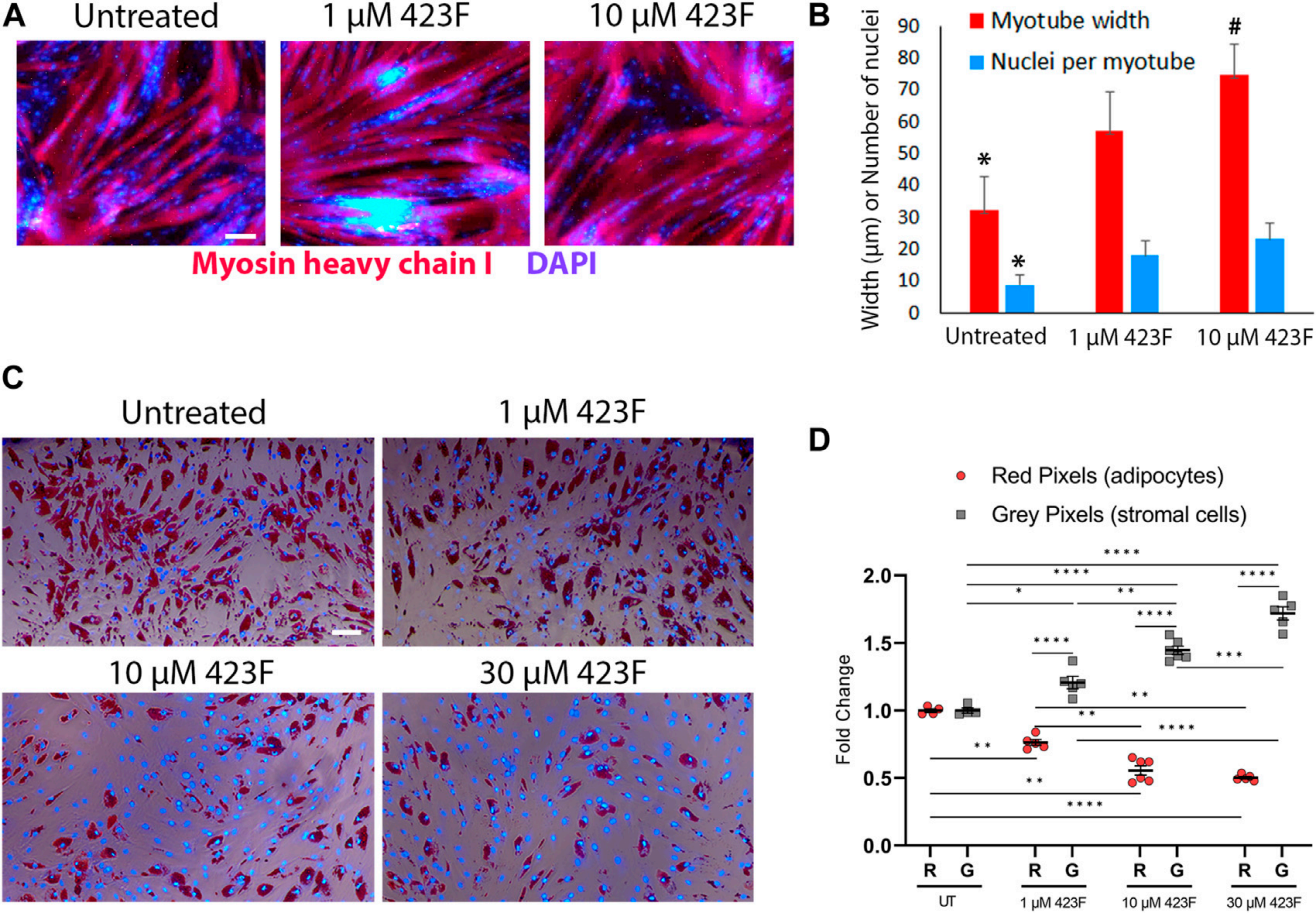
Different effects of 423F treatment on differentiated muscle progenitor derivatives. **(A, B)** Analysis of MyHC I^+^ myotube maturation based on myotube diameter and number of nuclei per myotube as seen in representative images of untreated and 423F treated cultures of CD56^+^CD90^+^ progenitors **(A)** and matched measurements **(B)**. Graphs represent mean ± SEM. n = at least 3 donors. Data analyzed via one-way ANOVA. *p < 0.001 between 423F treated cultures and untreated control cultures, #*p* < 0.0000001 between 1 µM treated cultures and 10 µM treated cultures. **(C, D)** Survival of human muscle-derived mature adipocytes (Oil red O^+^DAPI^+^ cells) and FAP (gray DAPI^+^ cells) in mixed cultures in the absence or presence of increasing concentrations of 423F. Blue nuclear staining for DAPI and Oil red O staining for adipocytic lipid droplets **(C)**. Percentage of red pixels (adipocytes) and gray pixels (FAP) out of total area of images of 423F treated cultures **(D)**. Data was analyzed via paired one-way ANOVA for comparisons between 423F concentrations between the same cell and unpaired Student’s *t* test for comparisons between adipocytes and stromal cells, followed by Tukey’s *post hoc* multiple comparisons. ****p < 0.0001, ***p < 0.001, **p < 0.01, *p < 0.05. Scale bars represent 100 μm.

Considering that administration of 423F can concurrently modulate the differentiation and cell composition of the progressively degenerating skeletal muscle, we further evaluated the effect of 423F/gp130 signaling on muscle progenitor differentiated progeny. To evaluate myotube growth (Hindi et al., 2013), we measured myotube diameter and quantified the number of nuclei per myotube in cultures of CD56^+^CD90^+^ myogenic subset in the presence or absence of 423F. Progenitor derived myotubes were significantly larger in 423F treated cultures than myotubes in untreated cultures 423F. Greater increase in myotube size was measured in the presence of higher 423F concentration than that of untreated control cultures or cultures treated with lower concentrations of 423F (Figures 8A, B). A maximal increase in myoblast fusion, which was indicated by the number of nuclei per myotube, was already achieved at lower concentrations of 423F (Figure 8B).

It was previously shown that IL-6 and STAT3 are required for adipocyte lipolysis (Guo et al., 2021; Gandhi et al., 2022). We therefore hypothesized that mature adipocytes may be more susceptible to 423F-induced modulation of gp130/STAT3 signaling rather than FAP. FAP were first induced to differentiate into adipocytes in adipogenic medium for 6 days. At 6 days post adipogenic induction, FAP-derived cultures were composed of mature Oil red O^+^ adipocytes and a fraction of non-differentiated FAP (Figure 8C). The effects of 423F on mature adipocytes were further tested in these mixed cultures. Mature adipocytes derived from human muscle FAP following 6 days of adipogenic induction were eliminated from 423F-treated cultures in a dose-dependent manner (Figures 8C, D). Conversely, 423F treatment did not affect the growth of non-differentiated muscle FAP in these mixed adipocytes-FAP cultures (Figures 8C, D). Taken together, these data demonstrate that 423F can simultaneously promote myo-regeneration and diminish chronic muscle degeneration by inhibition of fibrosis progression as well as selective elimination of developing intramuscular adipose tissue.

## Discussion

Although primary cells deliver more relevant outcomes than cell lines, derivation of cultured primary cells from different donors or various muscle types selected for biopsy might increase the variability of experimental outcomes, as they respond differently to culture inductions. Additionally, the characteristics of primary cells change with each subsequent passage (Goloviznina et al., 2020; Testa et al., 2020). Combined, our studies address these experimental challenges and present a novel human muscle progenitor-based platform that allows examination of the potential therapeutic effects of a candidate drug in an environment of multiplexed signals. CD56 and CD90 markers were used to identify three distinct muscle progenitor subsets that maintained their intrinsic differentiation potential in single and multiplexed culture conditions: myogenic, non-fibro-adipogenic CD56^+^CD90^+^ subset, non-myogenic, non-adipogenic CD90^+^CD56^−^ subset and CD90^−^CD56^−^ subset that demonstrated vast fibro-adipogenic differentiation but no myogenic differentiation in any tested conditions. Additionally, this system allowed us to elicit major differences in 423F/gp130 modulated STAT3 activation, differences that directly correlated with the lineage of differentiation. Finally, 423F was shown to mediate inhibition of both fibrogenesis and adipogenesis while mediating an increase in myogenesis, suggesting its use as a potential triple therapeutic drug.

Significant progress is evident in the recent development of advanced *in vitro* models intended for the study of muscle development, differentiation, and diseases. This includes 3D mixed cultures of primary-muscle-derived progenitors and vascular/neuronal cells, as well as organoids and pluripotent stem cell myogenic derivatives (Jalal et al., 2021). Surprisingly, muscle cell response to mixed and/or opposing signaling that operate at the same time has not been identified so far, especially since it is most likely that these are the conditions in remodeling muscle. For example, continuous induction of T cells in mixed culture conditions revealed a continuum of T cell fate with mixed phenotype and altered cytokine expression patterns (Antebi et al., 2013). Such experimental systems contribute to validation of theoretical models aimed to predict cell fates and facilitate the development of improved theoretical models of cell differentiation network and identification of intervention targets *in silico* (Zanudo and Albert, 2015; Faustino et al., 2022).

While mixed inductions, as we use here, do not reveal the complete intricacies of the regulatory networks controlling cell differentiation, they are still valuable tools for monitoring the complexity and potential therapeutic effects of a tested drug in the settings of chronic muscle injury. Our model can be refined when more parameters, such as extracellular matrix of healthy and diseased muscle, are integrated into the reporter system. Moreover, sequential stepwise combinations of myo-fibro-adipogenic inductions may reveal continuous, terminal, or reversed differentiation of various muscle progenitor subsets.

Although human mesodermal precursors were extensively studied and retrospectively identified in culture systems since the 90s (Caplan, 1991; Pittenger et al., 2019), their differentiation capability is still characterized in single traditional induction cultures using highly identified regulators known to push mesodermal cells into a particular cell fate. However, an alternative probabilistic culture model should predict differentiation capability of a tested cell population under mixed conditions that are more likely to resemble the conditions in remodeling injured tissue. So far, cell differentiation response to multiplexed signal or multi-factor network reprogramming were studied empirically only in hematopoietic cell subsets, such as naïve T cells, as well as cancer cells and matched tumor derived mesodermal cells (Antebi et al., 2013; Zanudo and Albert, 2015; Shamai et al., 2019), but not in human or mouse muscle precursors. Our findings reveal that cell fate is more dominantly regulated by intrinsic properties than external signals, and that fibrogenic signaling input dominates and eliminates any degree of adipogenic differentiation when opposing pathways are simultaneously activated in fibro-adipogenic cultures. This observation is compatible with the histological analysis of degenerating RC muscles and corresponds to the presence of terminally differentiated scar populating myofibroblasts or adipose-tissue-residing mature adipocytes, but absence of cells with combined fibro-adipogenic appearances (Jensen et al., 2018; Sharma et al., 2020; Wu et al., 2021).

IL-6 signaling was shown to mediate pleiotropic effects in muscle via modulation of STAT3 activity. While constitutive activation of STAT3 is linked to cancer cell proliferation and survival (Haura et al., 2005), enhanced proliferation of mouse and human cells is promoted by inhibition or knockdown of STAT3 activity *in vitro* and *in vivo* (Price et al., 2014; Tierney et al., 2014). Adding to the complexity of STAT3-mediated regulation of myogenic cell responses, transient activation of STAT3 is crucial for pro-myogenic (Wang et al., 2008) and final myogenic differentiation (Tierney et al., 2014). Moreover, STAT3-mediated intrinsic regulation of myogenic features of satellite cells in both aged and dystrophic muscle (McKay et al., 2013; Price et al., 2014; Tierney et al., 2014). Our studies in human skeletal muscle primary progenitors extend findings in aged and genetically abnormal satellite mouse cells to human progenitors with normal genetic background.

Finally, we demonstrate here that TGFβ-1 signaling is sufficient to eliminate adipogenic potential of FAP in dual and triple culture conditions. However, adipogenic conditions did not affect TGFβ-1 induced increase in collagen production in AF and AFM conditions. Therefore, finding a drug that can target two intracellularly linked opposing pathological differentiation pathways independently of each other is critical. This is especially important when inhibition of one degenerative process shifts the balance and drives the other degenerative process, and by that preventing one type of damage but worsening the other. For example, the existence of such functional crosstalk was discovered when the expression of WISP-1 was modulated in perivascular progenitor cells, wherein WISP-1 positively regulated osteogenesis at the expense of adipogenesis (Meyers et al., 2018). Likewise, PDGFRα signaling directs fat-tissue-residing nestin^+^ perivascular cells towards fibrogenic differentiation, which, in turn, opposes adipogenesis (Iwayama et al., 2015). Another more clinically relevant study revealed that, similar to our findings, CD90^+^ FAP from degenerating muscle of type-2 diabetic patients were less adipogenic and switched to a pro-fibrogenic instead of adipogenic phenotype following metformin treatment. Moreover, collagen I expression levels correlate with PDGFRα expression and signaling in these cells (Farup et al., 2021). Our studies demonstrate that it is possible to bypass this shift in direction of differentiation balance by modulating the activity of a common target protein, and that the resulting therapeutic effects might be exclusively type-of-disease-dependent. Combined data from several separated publications further support this concept, with the first study demonstrating and defining the role of bone morphogenic protein 3b (BMP3B), also known as growth differentiation factor 10 (GDF10), as anti-fibrotic. Bmp3b-null fibroblasts were more susceptible to TGF-β–induced fibrogenic changes and administration of BMP3B was effective against TGF-β-induced fibrogenesis in lung fibroblasts, especially in the suppression of excessive extracellular matrix production (Suzuki et al., 2022). The second study highlighted the protective role of BMP3B against sarcopenia via preservation of muscle mass and innervated neuromuscular junctions, as well as inhibition of fibro-adipogenic differentiation of FAP in aged mice (Kurosawa et al., 2021; Uezumi et al., 2021). A third study showed that BMBP3/GDF10 secreted from the CD142^+^ subpopulation of mouse and human muscle progenitors represses the adipogenic differentiation of CD142^-^ cells (Camps et al., 2020). Therefore, in the context of pharmacotherapy, drug-mediated inhibition of fibrosis could shift the balance towards increased adipogenesis. Thus, it seems essential to validate the disease modifying activity of a candidate drug when multiple degeneration pathways are activated, particularly when a functional crosstalk between fibrogenic, adipogenic, and osteogenic signaling regulates FAP fate. Altogether, our work indicates that modulation of gp130 signaling affects the differentiation of distinct human muscle cell subsets differently. Future experiments are designed to elucidate signaling pathways and complete a comparative study of equivalent cell subsets from diseased human muscles.

## Data Availability

The raw data supporting the conclusion of this article will be made available by the authors, without undue reservation.
