# Supplementary material for: Distinct human skeletal muscle-derived CD90 progenitor subsets for myo-fibro-adipogenic disease modeling and treatment in multiplexed conditions
Source: Front Cell Dev Biol. 2023 Apr 18;11:1173794. doi: 10.3389/fcell.2023.1173794 (PMC10151706; doi:10.3389/fcell.2023.1173794)
Supplement: Supplementary file 1 [file DataSheet1.pdf]

Supplementary Material

Distinct human skeletal muscle-derived CD90 progenitor subsets for myo-fibro-adipogenic disease modeling and treatment in multiplexed conditions

Angela Li[1], Madhavan Anbuchelvan[1], Amir Fathi[1], Maya Abu-Zahra[1], Denis Evseenko[1,2], Frank A. Petrigliano[1†], and Ayelet Dar[1*†]

[1]Department of Orthopaedic Surgery, Keck School of Medicine, University of Southern California, Los Angeles, CA, United States.
[2]Department of Stem Cell Research and Regenerative Medicine, University of Southern California, Los Angeles, CA, United States.

*** Correspondence:**
Ayelet Dar
darayelet@gmail.com

†These authors share senior authorship.

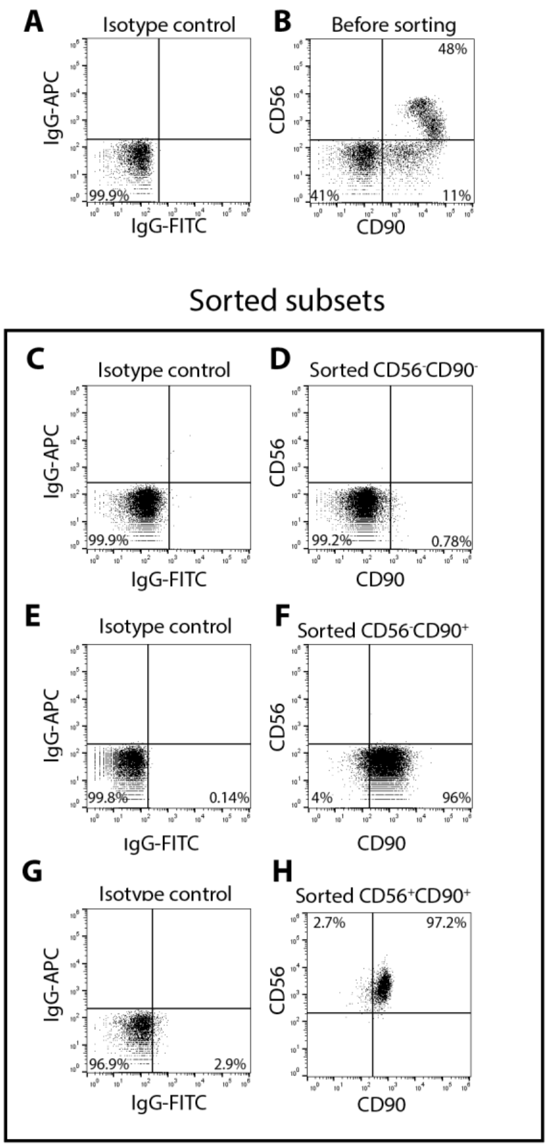

Supplementary Figure S1. Representative dot plots of flow cytometry analysis and sorting of CD56/CD90 human muscle-derived subsets and matched isotype controls. (**A-B**) Flow cytometry analysis reveals the presence of three distinct subsets, CD56$^+$CD90$^+$, CD56$^-$CD90$^+$ and CD56$^-$CD90$^-$**,** derived from cultures of human muscle between passage 0 and passage 1. (**C-H**) Subsets were sorted as indicated (D, F, H), further cultured and analyzed for the expression of CD56 and CD90 between passage 1 to passage 2 (**D, F, H**). **A, C, E, G** represent matched isotype controls.

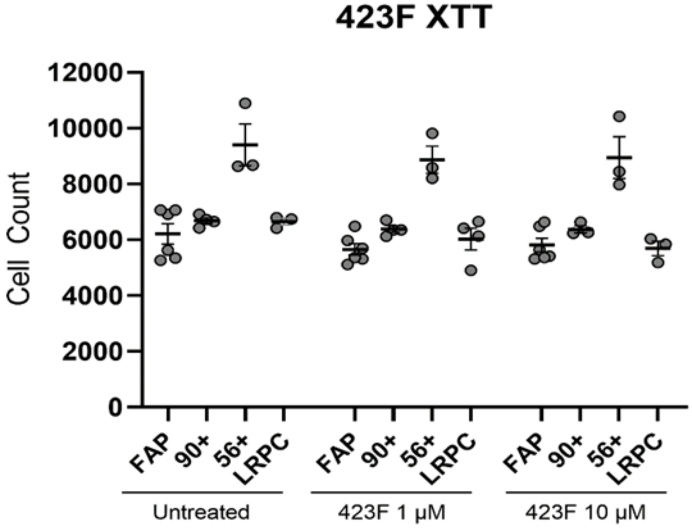

Supplementary Figure S2. Assessment of cell viability and proliferation of 423F treated and untreated human muscle cell subsets by XTT assay. Colorimetric quantification of culture metabolism of undifferentiated FAP, CD56, CD90 and LRPC treated with 1 μM and 10 423F μM for 48 hours. Data are presented as mean ± SD (one-way ANOVA).

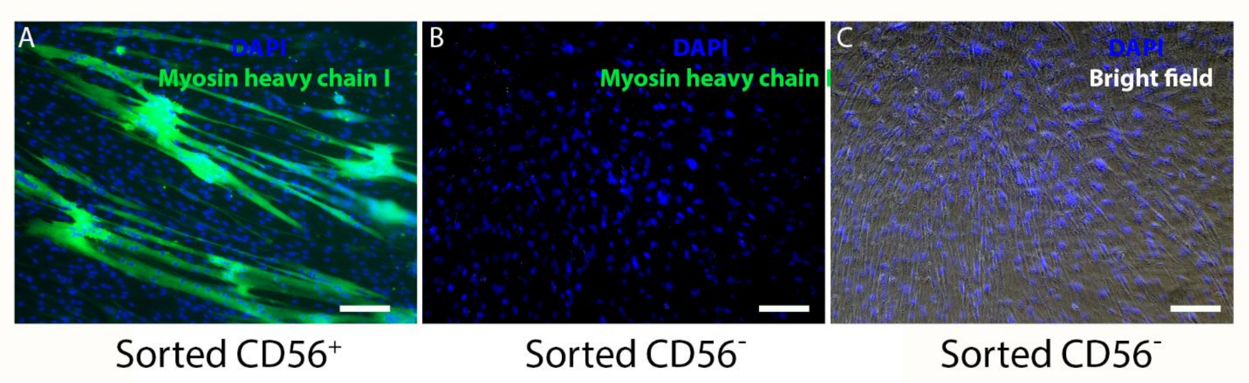

Supplementary Figure S3. Representative images of human muscle sorted $CD56^{+}$ and $CD56^{-}$ subsets in Myogenic cultures. Enzymatically digested human muscle biopsies were sorted between passage 0 to passage 1. (**A**) Only $CD56^{+}$ subset exhibited myogenic differentiation as indicated by the presence of multinucleated (DAPI, blue), myosin heavy chain I^{+} (green) myotubes within 6 days post induction. (**B-C**) $CD56^{-}$ fraction did not express myosin heavy chain I (**B**) and did not generate multinucleated (DAPI, blue**, C**) myotubes in myogenic cultures. Scale bar 50 μm.

Supplementary Table S1. Human healthy and diseased skeletal muscle biopsies included for analysis.

Sample	Age	Gender	Healthy muscle
D1	81	Male	Pectoralis, subscapularis
D2	30	Male	Deltoid
D4	65	Male	Deltoid
D5	51	Female	Deltoid
D6	73	Male	Left Subscapularis. Deltoid
D7	77	Female	Right shoulder Deltoid
D8	75	Male	Right Pectoralis
D9	33	Male	Quadriceps
D10	70	Male	Left Deltoid
D11	65	Male	Deltoid
D12	20	Male	Quadriceps
D13	44	Male	Deltoid
**Sample**	**Age**	**Gender**	**Diseased muscle**
Da	61	Male	Rotator cuff
Db	79	Female	Deltoid
Dc	68	Female	Rotator cuff

Supplementary Table S2. List of antibodies

**Immunolabeling**<br><br>**Name**	**Company**<br><br>(dilution)	**Flow cytometry (anti-human)**<br><br>**Name**	**Company**
Rabbit anti-mouse/human PDGFRβ	Cell Signaling (1:100)	PDGFRβ-PE, clone 28D4	BD
Mouse anti-human PDGFRβ	Abcam (1:50)	PDGFRα-PE, clone αR1	BD
Rabbit anti-mouse/human PDGFRα	Cell Signaling (1:1000)	CD146-PE, clone P1H12	BD
Goat anti-human PDGFRα	R&D (1:50)	CD34-APC, clone 581	BD
Rabbit anti-human CD90	NovusBio (1:50)	CD9-FITC, clone HI9A	Biolegend
Mouse anti-mouse/human vWF	NovusBio (1:1000)	CD15-FITC, clone HI98	Biolegend
Mouse anti-human MyHC	R&D (1:100)	CD90-FITC, clone 5E10	Biolegend
Donkey anti-mouse IgG Alexa-647	Invitrogen (1:400)	CD73-FITC, clone AD2	Biolegend
Donkey anti-mouse IgG Alexa-568	Invitrogen (1:400)	CD56-APC, clone 5.1H11	Biolegend
Donkey anti-rabbit IgG Alexa-555	Invitrogen (1:400)	Mouse IgG2a or IgG1, κ PE	Biolegend
Donkey anti-goat IgG Alexa-488	Invitrogen (1:400)	Mouse IgG1 IgG2a, κ FITC	Biolegend
Donkey anti-mouse IgG Alexa-647	Invitrogen (1:400)	Mouse IgG1 or IgG2a, κ APC	Biolegend
